# Predictive role of ventricular repolarization parameters for the occurrence of complete heart block in patients undergoing transcatheter aortic valve implantation

**DOI:** 10.1111/anec.12734

**Published:** 2019-12-06

**Authors:** Erdem Karacop, Asim Enhos

**Affiliations:** ^1^ Department of Cardiology Faculty of Medicine BezmiÂlem Foundation University Istanbul Turkey

**Keywords:** complete atrioventricular block, transcatheter valve implantation, ventricular repolarization

## Abstract

**Background:**

We investigated the role of ventricular repolarization parameters to predict complete atrioventricular block in patients undergoing transcatheter aortic valve implantation (TAVI).

**Methods:**

A total of 150 patients undergoing TAVI due to severe aortic stenosis were included in this retrospective cohort study. Patients were assigned in two groups based on the presence (*n*: 49) or absence (*n*: 101) of complete atrioventricular block after TAVI. Ventricular repolarization intervals (QT, QTc, JT, JTc, TP‐E), indices (QT dispersion), and ratios (TP‐E/QT, TP‐E/QTc, TP‐E/JTc) were measured.

**Results:**

Electrocardiographic repolarization parameters such as Tp‐e interval: 74.2 ± 5.1 versus 59.2 ± 6.1 ms, *p* < .001; QTc interval: 397.6 ± 3.4 versus 368.1 ± 7.8 ms, *p* < .001; JTc interval: 317.4 ± 11.3 versus 291.1 ± 6.7 ms, *p* < .001; Tp‐e/QT ratio: 0.28 ± 0.04 versus 0.20 ± 0.04, *p* < .001; Tp‐e/QTc ratio: 0.29 ± 0.04 versus 0.19 ± 0.03, *p* < .001; Tp‐e/JT ratio: 0.32 ± 0.03 versus 0.23 ± 0.03, *p* < .001; Tp‐e/JTc ratio: 0.30 ± 0.02 versus 0.2 ± 0.03, *p* < .001; and QT dispersion: 34.4 ± 3.0 versus 17.8 ± 3.6 ms, *p* < .01 were significantly higher in post‐TAVI permanent pacemaker group. In a univariate regression analysis, pulmonary artery pressure, heart rate, coronary artery disease, Tp‐e/QTc, Tp‐e/JTc, and PR interval were significantly associated with complete heart block. Tp‐e/JTc (OR 0.373, *p* = .067) and PR interval (OR 0.898, *p* = .079) were found to be independent predictors of these type of arrhythmias in a multivariate analysis. But it is not statistically significant.

**Conclusion:**

Our results suggest that repolarization parameters may play a role in predicting complete atrioventricular block. Tp‐e/JTc was found to be potential independent risk marker for this setting.

## 1 INTRODUCTION

Transcatheter aortic valve implantation (TAVI) has emerged as a standard therapy for inoperable, high‐risk, and intermediate‐risk patients with low mortality and complication rates (Linke et al., 2014; Popma et al., 2014). Trials examining the efficacy and safety of TAVI in low‐risk patients are currently ongoing. TAVI has been increasingly performed with a minimalist approach, and the rate of periprocedural complications has decreased over time (Reinöhl et al., 2015; Vahl, Kodali, & Leon, 2016). This resulted in an extension of TAVI indications to treating individuals at lower surgical risks (Leon et al., 2016; Thourani et al., 2016).

The incidence of conduction disturbances (complete heart block) requiring permanent pacemaker implantation has failed to decrease in recent times unlike other procedural complications. While 8.5%–25.9% of patients undergoing TAVI require a permanent pacemaker (PPM) depending on the type of prosthesis within 30 days (Erkapic et al., 2012), only 7% of patients undergoing surgery need a PPM after the procedure (Mohr et al., 2014). Predictors of post‐TAVI PPM implantation can be electrocardiographic, patient‐related, and procedural factors. Pre‐existing right bundle branch block, periprocedural atrioventricular (AV) block, prolonged QRS duration, longer PR at baseline, and pre‐existing left anterior fascicular block are reported as electrocardiographic factors. Among these, pre‐existing right bundle block is the most predictive one (Erkapic et al., 2012).

Patients with aortic stenosis generally have compensatory hypertrophy and interstitial fibrosis of the left ventricle. Abnormal ventricular repolarization may be identified in electrocardiography (ECG) recordings of patients with aortic stenosis as a result of myocardial fibrosis. Ventricular repolarization is a complex electrical phenomenon, which can be detected easily on 12‐derivation ECG recordings. QT interval is composed of both depolarization and repolarization phases, and it is also affected by QRS period. Because of unique reflection potential of ventricular repolarization, JT interval is a more specific repolarization marker than the QT interval (Bihlmeyer et al., 2018). Tp‐e interval, which is a relatively new ECG parameter, shows the interval between the peak and the end of T wave. However, Tp‐e interval is influenced by both heart rate and body weight. Therefore, Tp‐e/QT, Tp‐e/QTc, and Tp‐e/JTc have been proposed to be beneficial and useful markers of ventricular repolarization.

To our knowledge, there was no clinical study in the literature, which has evaluated the predictive value of ventricular repolarization markers on ECG for possibility of post‐TAVI PPM implantation due to complete heart block in patients undergoing TAVI procedure. Hence, we aimed to analyze the ventricular repolarization phase of patients with aortic stenosis in order to investigate for any predictive role for complete heart block. Moreover, we have considered various electrocardiographic intervals (QT, QTc, JT, JTc, TP‐E), indices (QT dispersion), and ratios (TP‐E/QT, TP‐E/QTc, TP‐E/JTc) that have been introduced for a detailed non‐invasive evaluation of the ventricular repolarization.

## 2 METHODS

### 2.1 Study population

Four hundred and ten patients who underwent transfemoral TAVI between 2013 and 2018 years in our clinic were analyzed retrospectively. CoreValve prosthesis (Medtronic) was implanted in 252 patients. Among these, 102 patients were not included in this analysis due to the presence of exclusion criteria. One hundred fifty patients were included. Study groups were divided into two groups as follows: 49 patients requiring post‐TAVI PPM due to complete heart block and 101 patients who underwent TAVR with no complete heart block. Exclusion criteria of the present study were atrial fibrillation, right bundle branch block, left bundle branch block, left anterior fascicular block, left posterior fascicular block, bifascicular block and nonspecific intraventricular conduction defects, previous pacemaker implantation, ECGs without clearly analyzable QT segment, type I and III antiarrhythmic usage, end‐stage hepatic, and renal failure. Baseline demographic and clinical characteristics of the patients were recorded from hospital records. Furthermore, ECG recordings were analyzed by two clinical cardiologists, who were unaware of the study results.

### 2.2 Electrocardiography

Standard 12‐lead ECG was recorded at 25 mm/s paper speed and 10 mm/mv amplitude in the supine position before TAVI, on post‐TAVI day 1 and before discharge for each patient. All ECG recordings were scanned and transferred to the digital platform. The QT interval was measured from the beginning of the QRS complex to the end of T wave and QTc for the heart rate using Bazett's formula QTc:qt√RR interval. The QTd was defined as the difference between the maximum and minimum QT interval of the 12 leads. Tp‐e interval was defined as the interval from the peak of a T wave to the end of T wave. Measurements of Tp‐e interval were performed from the precordial leads, and lead V_2_ was selected for measuring. Tp‐e/QT and Tp‐e/QTc ratios were calculated as the ratio of Tp‐e to the corresponding QT and QTc interval in lead V_2_. JT intervals were measured from the end of the QRS complex (J point) to the end of the T wave (JT end interval). JTc was calculated by using Hodge's formula [JTc:JT + 0.00175*(HR‐60)]. Intraobserver difference for JT, QT, and Tp‐e measurements was 2.8%, 2.3%, and 2.5%. This systematic error was similar for both groups.

### 2.3 Echocardiography

Transthoracic 2‐D echocardiography was performed for each patient before TAVI and repeated on post‐TAVI day 1 and 3 months after TAVI by using commercially available equipment (VIVID 7 Dimension Cardiovascular Ultrasound System) (Vingmed‐General Electric) with a 3.5 MHz transducer. LV diameters were measured using M‐mode imaging and left atrial area in the four‐chamber apical view. Ejection fraction was calculated by using modified Simpson biplane method. Assessment of aortic stenosis (AS) severity was based on both aortic mean gradient and aortic valve area. Aortic jet velocity was calculated by Doppler echocardiography. AS was defined as mild if mean systolic transaortic gradient was less than 25 mm Hg or jet velocity was between 3.0 and 4.0 m/s and severe if mean systolic transaortic gradient was greater than 40 mm Hg or jet velocity was greater than 4.0 m/s. All echocardiographic examinations were performed by an experienced cardiologist.

### 2.4 Device and procedure

Transcatheter aortic valve implantation procedure was performed under general anesthesia using a transfemoral approach. The Amplatz Extra Stiff Guide Wire was advanced to the apex of the left ventricle using 16‐F sheath through the femoral artery. A balloon valvuloplasty was applied on the aortic valve with ventricular pacing at a rate of 80–200 beats/min. Under fluoroscopic guidance, CoreValve prosthesis (Medtronic) was inserted into the native aortic annulus. After achievement of optimal opening, aortic root, aortic valve, and pericardium were visualized. Dual antiplatelet therapy (100 mg acetylsalicylic acid plus 75 mg clopidogrel) was administered to all patients for 6 months following TAVI.

### 2.5 Statistical analysis

Shapiro–Wilk test was used for assessing whether the variables follow normal distribution or not. Quantitative variables are expressed as mean + standard deviation (*SD*), and qualitative variables are expressed as numbers and percentages. Differences between independent groups were assessed by Student's *t* test for normally distributed quantitative variables, Mann–Whitney *U* test for variables without normal distribution, and chi‐square for qualitative variables. A *p* value lower than .05 was considered as statistically significant.

## 3 RESULTS

The study population was categorized into two groups according to post‐TAVI PPM requirement due to complete heart block as control TAVI group (*n* = 101) and post‐TAVI PPM group (*n* = 49). Baseline characteristics of the study groups were presented in Table 1. There was no difference between two groups in terms of age, gender, hypertension, hyperlipidemia, diabetes mellitus, chronic obstructive pulmonary disease, peripheral vascular disease, past history of cerebrovascular event, and smoking status (*p* > .05). Past history of coronary artery disease was higher in the post‐TAVR PPM group as compared to the control group (*p* < .05). Echocardiographic data and both the logistic EURO and STS scores were similar between two groups (*p* > .05).

**Table 1:** Baseline characteristics of two groups

Baseline characteristics	Control TAVI group (*n* = 101)	Third degree AV block‐TAVI (+) (*n* = 49)	*p* Value
Age (mean)	80.36 ± 8.7	81.84 ± 7.0	.3
Male/female	74/27	35/14	.5
Hypertension	83 (82%)	35 (70%)	.28
Diabetes mellitus	36 (35%)	16 (32%)	.4
Hyperlipidemia	21 (20%)	4 (8%)	.09
Coronary artery disease	38 (37%)	30 (60%)	<.05
Smoke	38 (37%)	13 (26%)	.6
Peripheral vascular disease	2 (2%)	5 (10%)	.2
Previous cerebrovascular event	7 (6%)	2 (4%)	.3
COPD	20 (19%)	13 (26%)	.7
LVEF (mean %)	53.10 ± 12.1	51.69 ± 13.8	.5
GFR (ml min^−1^ 1.73 m^−2^)	53.95 ± 20.1	54.39 ± 21.8	.9
Pulmonary artery pressure (mm Hg)	36.64 ± 12.1	44.33 ± 16.3	.8
Logistic EuroSCORE (mean ± *SD*) %	21.5 ± 2.8	20.9 ± 3.3	.1
STS score (mean ± *SD*) %	10.0 ± 3.5	9.9 ± 4.2	.7

Abbreviations: COPD, chronic obstructive pulmonary disease, GFR, glomerular filtration rate, LVEF, left ventricle ejection fraction.

Compared with control group, electrocardiographic repolarization parameters were significantly higher in the post‐TAVR PPM group (Tp‐e interval: 74.2 ± 5.1 vs. 59.2 ± 6.1 ms, *p* < .001; QTc interval: 397.6 ± 3.4 vs. 368.1 ± 7.8 ms, *p* < .001; JTc interval: 317.4 ± 11.3 vs. 291.1 ± 6.7 ms, *p* < .001; Tp‐e/QT ratio: 0.28 ± 0.04 vs. 0.20 ± 0.04, *p* < .001; Tp‐e/QTc ratio: 0.29 ± 0.04 vs. 0.19 ± 0.03, *p* < .001; Tp‐e/JT ratio: 0.32 ± 0.03 vs. 0.23 ± 0.03, *p* < .001; Tp‐e/JTc ratio: 0.30 ± 0.02 vs. 0.22 ± 0.03, *p* < .001; QT dispersion: 34.4 ± 3.0 vs. 17.8 ± 3.6 ms, *p* < .01) (Table 2).

**Table 2:** Electrocardiographic findings of the study population

	Control TAVI group (*n* = 101)	Complete heart block‐TAVI (+) (*n* = 49)	*p* Value
Heart rate (bpm)	73.6 ± 8.9	87.1 ± 8.6	<.001
Tp‐e (ms)	59.2 ± 6.1	74.2 ± 5.1	<.001
QTmin (ms)	349.0 ± 3.8	349.3 ± 3.9	.669
QTmax (ms)	374.8 ± 5.4	374.7 ± 5.6	.912
QTc (ms)	368.1 ± 7.8	397.6 ± 3.4	<.001
JT (ms)	286.0 ± 4.4	286.1 ± 4.4	.874
JTc (ms)	291.1 ± 6.7	317.4 ± 11.3	<.001
Tp‐e/QT	0.20 ± 0.04	0.28 ± 0.04	<.001
Tp‐e/QTc	0.19 ± 0.03	0.29 ± 0.04	<.001
Tp‐e/JT	0.23 ± 0.03	0.32 ± 0.03	<.001
Tp‐e/JTc	0.22 ± 0.03	0.30 ± 0.02	<.001
QTd (ms)	17.8 ± 3.6	34.4 ± 3.0	<.001
QRS (ms)	85.9 ± 3.8	85.8 ± 3.9	.943

Note

JT interval (JT) was measured from the end of the QRS complex (J point) to the end of the T wave (JT end interval); QT dispersion (QTd) was determined as the difference between the maximum and minimum QT interval.

Abbreviations: bpm, beat per minute; JTc, corrected JT interval; ms, millisecond; QTc, corrected QT interval; QTmax, maximum QT; QTmin, minimum QT; Tp‐e, T peak and end interval.

**Figure 1:**
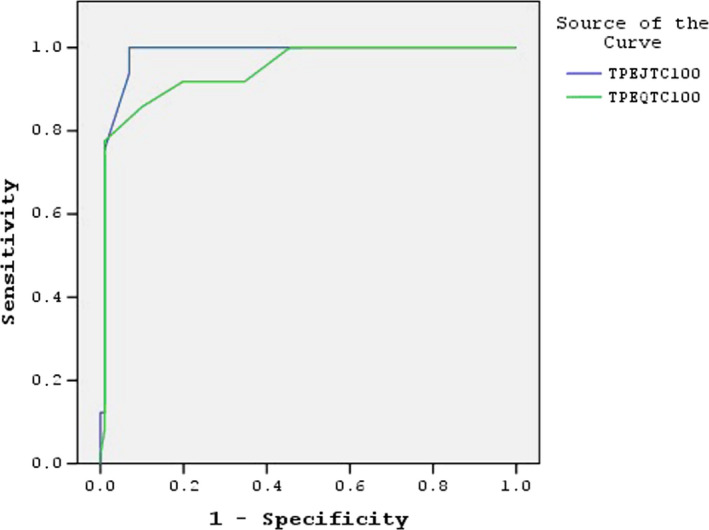
Receiver operating characteristic curve of TP‐E/JTc × 100 and TP‐E/QTc × 100

A receiver operating characteristic curve was generated for sensitivity and specificity, and there spective areas under the curve (AUCs) were used to investigate the diagnostic value of ventricular repolarization parameters for complete heart block in patients undergoing TAVI procedure (Figure 1). The analysis indicated that Tp‐e/QTc >25 had a 85.7% sensitivity and 90.1% specificity for complete heart block (AUC 0.947, 95% CI 0.909–0.984; *p* < .001). Their negative and positive predictive values were 86.3% and 89.6%, respectively. Tp‐e/JTc levels >27.5 had 93.9% sensitivity and 93.1% specificity (AUC 0.982, 95% CI 0.963–1.002; *p* < .001). Their negative and positive predictive values were 93.8% and 93.1%, respectively.

PR interval was significantly higher in the post‐TAVI PPM group (194.00 ± 31.3 vs. 136.07 ± 20.12 ms, *p* < .001) (Table 3). There was no difference between groups in terms of P wave amplitude and duration (*p* > .05).

**Table 3:** P wave amplitude, P wave duration, and PR interval

	Control TAVI group (*n* = 101)	Complete heart block‐TAVI (+) (*n* = 49)	*p* Value
P‐amplitude (mV)	0.13 ± 0.04	0.13 ± 0.04	.89
P‐duration (ms)	105.8 ± 15.4	106.0 ± 13.0	.95
PR interval (ms)	136.07 ± 20.12	194.00 ± 31.3	<.001

Abbreviations: ms, millisecond; mV, millivolt.

In a univariate analysis, pulmonary artery pressure, heart rate, coronary artery disease, Tp‐e/QTc, Tp‐e/JTc, and PR interval were significantly associated with the presence of complete heart block in TAVI patients. Tp‐e/JTc (OR 0.373, *p* = .067) and PR interval (OR 0.898, *p* = .079) were found to be independent predictors of these types of arrhythmias in a multivariate analysis, after adjusting for other risk parameters. But it is not statistically significant (Table 4).

**Table 4:** Univariate and multivariate regression analyses of predictors of complete heart block in patients undergoing TAVI

Variables	Univariate analysis		Multivariate analysis	
	Odds ratio (95% CI)	*p*	Odds ratio (95% CI)	*p*
Age	1.023 (0.980–1.068)	.304		
Gender	0.912 (0.426–1.951)	.813		
PAP	1.040 (1.014–1.067)	.002	0.851 (0.642–1.128)	.262
Heart rate	1.164 (1.108–1.222)	<.001	0.938 (0.799–1.101)	.433
CAD	2.168 (1.298–5.280)	.007	0.030 (0.001–2.400)	.117
PVD	1.051–30.116	.536		
TPEQTC × 100	1.608 (1.380–1.875)	<.001	0.836 (0.603–1.159)	.282
TPEJTC × 100	3.253 (2.034–5.203)	<.001	0.373 (0.130–1.072)	.067
P‐duration	1.001 (0.978–1.024)	.954		
PR interval	1.075 (1.051–1.099)	<.001	0.898 (0.796–1.012)	.079

Abbreviations: CAD, coronary artery disease; PAP, pulmonary artery pressure; PVD, peripheral artery disease; Tp‐eJTC, T peak and end interval JTC; Tp‐eQTC, T peak and end interval QTC.

## 4 DISCUSSION

In this study, we investigated the predictive potential of ventricular repolarization parameters, which can be detected on baseline ECG in terms of an occurrence of complete heart block in patients undergoing TAVI procedure. Main findings of the present study are as follows: (a) The electrocardiographic repolarization parameters (Tp‐e, QTc, JTc interval, Tp‐e/QT, Tp‐e/QTc, Tp‐e/JT, Tp‐e/JTc ratio, QT dispersion) were significantly higher in post‐TAVI PPM group as compared to the control TAVI group, (b) Tp‐e/JTc was found to be as significant as PR interval for prediction of complete heart block after adjusting for other risk parameters in patients undergoing TAVI procedure, and (c) Tp‐e/JTc was detected as potential marker, which may be used to predict the emergence of the complete atrioventricular arrhythmias in this setting, with strong sensitivity and specificity.

In severe aortic stenosis, myocardial hypertrophy is induced due to increased afterload and wall stress in a left ventricle. For that reason, wall thickness increases and myocytes enlarge to stabilize left ventricular function (Carabello, 1995; Grossman, Jones, & McLaurin, 1975). Left ventricle is unable to cope with an increased pressure afterload and eventually results in heart failure (Dweck, Boon, & Newby, 2012). Myocyte apoptosis and fibrosis increase as a result of this vicious cycle (Bishopric, Andreka, & Slepak, 2001; Dweck et al., 2011). Fibrosis damages conduction system and makes left ventricle to become more susceptible to arrhythmias (Dweck et al., 2012; Nerheim, Krishnan, & Olshansky, 2001). Additionally, fibrosis of the conduction system accounts for about one‐half of cases of the AV block.

The prevalence of hypertension is higher in patients with severe aortic stenosis, and it contributes to the development of myocardial hypertrophy and fibrosis (Hueb et al., 2011; Pate, 2002). It is also reported that hypertension causes remodeling on LV myocardium. Finally, remodeling causes abnormal ventricular repolarization. Tp‐e interval and Tp‐e/QT ratio were found to be correlated significantly with increased LV hypertrophy and LV remodeling in patients with hypertension (Ciobanu et al., 2017). In our study, one possible effect of fibrosis and remodeling, and some ventricular repolarization parameters such as Tp‐e/QTc and Tp‐e/JTc were found to be significantly higher in arrhythmic TAVI group as compared to control.

Common pathophysiology is remodeling and fibrosis of myocytes and conduction system. Myocardial fibrosis has a negative impact on prognosis in aortic stenosis and other cardiac pathologies. Varying degrees of interstitial fibrosis and impaired myocyte ultrastructure are characteristical pathological findings for AS. Degenerative changes, which lead to replacement or scarring fibrosis in conjunction with myocyte loss, starting in the subendocardial space spread to whole myocardium over time. It was shown that Tp‐e interval and Tp‐e/QTc ratios were significantly increased in patients with AS (Yayla et al., 2016). Additionally in a study by Kahraman et al. (2018), Tp‐e interval, Tp‐e dispersion, Tp‐e/QT ratio, and Tp‐E/QTc ratio were significantly higher in patients with AS than the control group as well as QT and QTc interval. In our study, we found an increase in ventricular repolarization markers, which were more pronounced in subgroup of severe aortic stenosis patients, in patients with complete heart block after TAVI. In addition to pre‐existing right bundle block, periprocedural AV block, prolonged QRS duration, longer PR at baseline, and pre‐existing left anterior fascicular block were reported as electrocardiographic predictors of post‐TAVI PPM in previous studies in the literature (Erkapic et al., 2012). Ventricular repolarization markers may play a role in predicting post‐TAVI PPM in severe aortic stenosis.

Transcatheter aortic valve implantation has been accepted as a novel advanced therapy for patients, who are in a intermediate–high‐risk group and inoperable for moderate‐to‐severe AS. Beyond this, the procedure has low mortality and complication rates when it is performed by experienced interventional cardiologists. During the past years, reduction in paravalvular leakage (PVL) had been of paramount importance to operators and industry (Binder et al., 2013; Meredith et al., 2014). The main reason for putting the focus on PVL rather than on new PPM is reduced survival in early trials (Leon et al., 2016; Toggweiler et al., 2013). SAPIEN 3 valve (Edwards Lifesciences) is more than double rates of PPM implantation than previous generation SAPIEN and SAPIEN XT valves (Wendler et al., 2017). Detrimental consequences of conduction disturbances and a decrease in left ventricular function induced by a right ventricle‐based paced rhythm are more considered as TAVI is set to expand to patients at intermediate and low surgical risk. In a recent retrospective study by Fadahunsi et al. (2016), patients from the Transcatheter Valve Therapy registry and CMS database showed 31% increase in risk of 1‐year mortality and 33% increase in risk of a composite of mortality and heart failure (HF) admissions at 1 year in patients, who underwent PPM implantation

In our study, coronary artery disease was significantly higher in the post‐TAVI PPM group (60% vs. 37%, *p* < .05). The atherosclerosis risk in communities study reported that QT interval prolongation (QTc ≥ 440 ms for men and QTc ≥ 454 ms for women) was associated with 1.5‐ to 5.0‐fold increased risk of incident coronary heart disease among general populations(Dekker, Crow, & Hannan, 2004) QT interval has also been shown to be associated with subclinical arterial disease(Festa et al., 1999). Coronary artery disease is very well known to be associated with abnormal ventricular repolarization parameters. Unfortunately, it may have a confounder effect in these parameters.

Discussion of post‐TAVI PPM implantation risk is paramount importance. Pre‐existing RBBB and a heavily calcified left ventricular outflow tract (LVOT) are most predictive of the need for post‐TAVI PPM implantation. New algorithm should be set to define the absolute risk for this clinical setting. In the present study, ventricular repolarization parameters (Tp‐e, QTc, JTc interval, Tp‐e/QT, Tp‐e/QTc, Tp‐e/JT, Tp‐e/JTc ratio, QT dispersion) were significantly higher in the post‐TAVI PPM group. It may be beneficial to make algorithms by adding ventricular repolarization parameters to predict adverse arrhythmic events after TAVI procedure. If the risk is high, precautions like high depth of implantation and using a balloon expandable valve may be taken into consideration.

### 4.1 Study limitations

There are some limitations to the present study. First, this is a retrospective study which arose from single center. Hence, short‐term follow‐up and long‐term follow‐up of the study patients were lacking. Second, relatively small sample population were enrolled in this study to clarify the relationship between ventricular repolarization parameters and post‐TAVI PPM implantation probability due to complete heart block in patients undergoing TAVI procedure.

## 5 CONCLUSIONS

This study is the first to demonstrate a predictive value of electrocardiographic ventricular repolarization parameters (Tp‐e, QTc, JTc interval, Tp‐e/QT, Tp‐e/QTc, Tp‐e/JT, Tp‐e/JTc ratio, QT dispersion) for complete heart block in patients with aortic stenosis undergoing TAVI. Moreover, at the end of our study, Tp‐e/JTc was found to be potential independent risk marker for this setting. Large multicenter prospective studies are needed to clarify the exact pathophysiological mechanism and relationship between these parameters on the ECG and the probability of complete atrioventricular block in TAVI patients.

## CONFLICT OF INTEREST

The authors declare that they have no conflict of interest. The authors alone are responsible for the content and writing of the paper. The authors have had full control of all primary data, and they agree to allow the journal to review their data if requested.

## AUTHORS' CONTRIBUTIONS

Conceived and designed the analysis: EK, AE

Collected the data: AE

Contributed data or analysis tools: EK

Performed the analysis: EK

Wrote this paper: EK

## ETHICS

All procedures performed in studies involving human participants were in accordance with the ethical standards of the institutional and/or national research committee and with the 1964 Helsinki declaration and its later amendments or comparable ethical standards.
